# Supplementary material for: Structural characterization of the microbial enzyme urocanate reductase mediating imidazole propionate production
Source: Nat Commun. 2021 Mar 1;12:1347. doi: 10.1038/s41467-021-21548-y (PMC7921117; doi:10.1038/s41467-021-21548-y)
Supplement: Supplementary file 1 — Supplementary Information [file 41467_2021_21548_MOESM1_ESM.pdf]

Supplementary information

Structural Characterization of the Microbial Enzyme Urocanate Reductase Mediating Imidazole Propionate Production

Raminta Venskutonytė[1], Ara Koh[2,3], Olof Stenström[4], Muhammad Tanweer Khan[2], Annika Lundqvist[2], Mikael Akke[4], Fredrik Bäckhed[2,5,6], Karin Lindkvist-Petersson[1,7*]

[1]Experimental Medical Science, Medical Structural Biology, BMC C13, Lund University, SE-221 84 Lund, Sweden

[2]Department of Molecular and Clinical Medicine/Wallenberg Laboratory, Institute of Medicine, University of Gothenburg, Gothenburg, Sweden

[3]Precision Medicine, Samsung Biomedical Research Institute, Samsung Medical Center, School of Medicine, Sungkyunkwan University (SKKU), Suwon, 16419, Republic of Korea

[4]Biophysical Chemistry, Center for Molecular Protein Science, Department of Chemistry, Lund University, Box 124, SE-221 00, Lund, Sweden

[5]Region Västra Götaland, Sahlgrenska University Hospital, Department of Clinical Physiology, Gothenburg, Sweden

[6]Novo Nordisk Foundation Center for Basic Metabolic Research, Section for Metabolic Receptology and Enteroendocrinology, Faculty of Health Sciences, University of Copenhagen, Copenhagen, Denmark

[7]LINXS - Lund Institute of Advanced Neutron and X-ray Science, Scheelevägen 19, SE-223 70, Lund, Sweden

[*]Corresponding author: karin.lindkvist@med.lu.se

Supplementary Table 1. Data collection and refinement statistics.

	uro-FAD	imp-FAD	apo-FAD	apo-ADP
**Data collection**				
Space group	P 31 2 1	P 31 2 1	P 6 2 2	P 21
Cell dimensions				
a, *b*, *c* (Å)	123.7, 123.7, 66.3	123.8, 123.8, 66.3	159.8, 159.8, 75.5	41.8, 95.8, 63.2
α, β, γ (°)	90, 90, 120	90, 90, 120	90, 90, 120	90, 91, 90
Resolution (Å)	45.23-1.56 (1.59-1.56)	45.25-1.40 (1.42-1.40)	46.13-2.56 (2.67-2.56)	63.12-1-10 (1.12-1.10)
R_{merge}	0.091 (1.704)	0.115 (1.630)	0.172 (0.593)	0.100 (0.698)
Mean I / σI	20.2 (2.1)	17.7 (2.5)	15.3 (2.6)	9.5 (1.2)
CC 1/2	1.00 (0.756)	0.999 (0.782)	0.996 (0.864)	0.999 (0.543)
Completeness (%)	100 (100)	100 (100)	98.1 (89.1)	98.6 (92.4)
Redundancy	20.2 (20.9)	21.2 (21.8)	18.8 (6.2)	8.8 (3.2)
**Refinement**				
Resolution (Å)	45.23-1.56	45.25-1.40	46.13-2.56	63.19-1.10
No. reflections	83061	114835	18292	198098
R_{work} / R_{free}	11.36/14.57	11.56/14.61	17.47/23.16	10.75/12.97
No. atoms				
Protein	7155	7194	3460	7473
FAD/ADP/ligand	84/-/15	84/-/17	53/-/-	-/39/-
Glycerol/sulfate/ chloride/sodium	111/25/2/1	154/-/4/1	18/30/3/1	42/-/5/-
Water	598	644	190	826
B-factors				
Protein	25.2	19.1	32.3	13.7
FAD/ADP/ligand	18.4/-/21.5	11.7/-/14.3	23.9/-/-	-/6.9/-
Glycerol/sulfate/ chloride/sodium	48.5/56.9/58.5/18.4	41.4/-/25.3/12.5	62.5/73.7/56.1/29.8	27.4/-/19.6/-
Water	36.7	34.3	33.5	27.9
R.m.s. deviations				
Bond lengths (Å)	0.008	0.010	0.004	0.008
Bond angles (°)	0.966	1.145	0.532	1.124
Ramachandran plot				
Favored (%)	97.79	98.01	97.79	97.59
Outliers (%)	0	0	0	0

Supplementary Note 1. Detailed expression for the model applied in ITC analyses. Derivation of model involving two coupled equilibria: protein dimerization and protein–ligand binding.

$[P]$, free protein concentration
$[L]$, free ligand concentration
$[PL]$, concentration of protein ligand complex
$[PP]$, concentration of protein dimer
P_{tot}, total protein concentration
L_{tot}, total ligand concentration
K_2, equilibrium constant of protein dimerization (association)
K_1, equilibrium constant of protein ligand binding (dissociation)
ΔH_1, enthalpy of ligand binding (association)
ΔH_2, enthalpy of protein dimerization (association)
V_0, reaction cell volume
V_i, injection volume
Q_i, heat function following the *i*th injection
ΔQ_i, heat released by the *i*th injection

$$PL \overset{K_1}{\leftrightarrow} P + L \quad (1)$$

$$2P \overset{K_2}{\leftrightarrow} \mathrm{PP} \quad (2)$$

$$K_1 = \frac{[P][L]}{[PL]} \quad (3) \qquad K_2 = \frac{[PP]}{[P]^2} \quad (4)$$

$$P_{tot} = [P] + 2[PP] + [PL] \quad (5)$$

$$L_{tot} = [L] + [PL] \quad (6)$$

$$P_{tot} = [P] + 2[PP] + [\mathrm{PL}] = 2[P]^2K_2 + [P] + \frac{[P][L]}{K_1} = 2[P]^2K_2 + [P] + \frac{[P]L_{tot}}{K_1 + [P]}$$

$$L_{tot} = [L] + \frac{[P][L]}{K_1} \Leftrightarrow [L] = \frac{L_{tot}}{1+\frac{[P]}{K_1}} = \frac{K_1L_{tot}}{K_1+[P]} \quad (7)$$

$$[PP] = [P]^2K_2 \quad (8)$$

$$[PL] = P_{tot} - [P] - 2[PP] \quad (9)$$

$$[L] = L_{tot} - [PL] \quad (10)$$

$$Q_i = \Delta H_1[PL]V_0 + \Delta H_2[PP]V_0 \quad (11)$$

$$\Delta Q_i = Q_i - Q_{i-1} + \frac{V_i}{V_0}\left(\frac{Q_i - Q_{i-1}}{2}\right) + Q_{off} \quad (12)$$

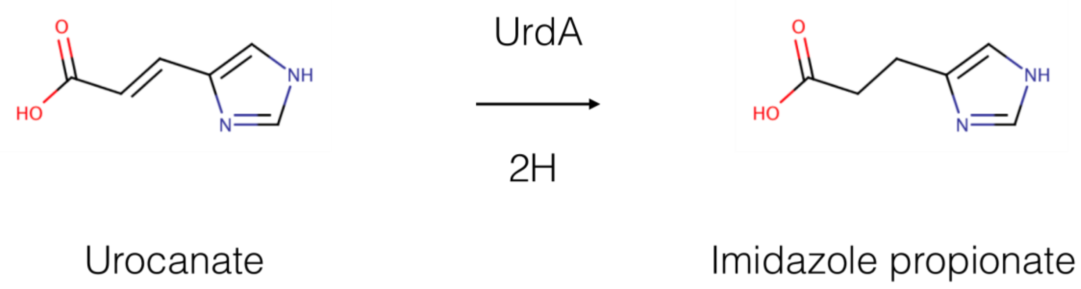

Supplementary Figure 1. UrdA catalyzes the reduction of urocanate to imidazole propionate. Compound structures prepared in MarvinSketch (version 19.12 ChemAxon).

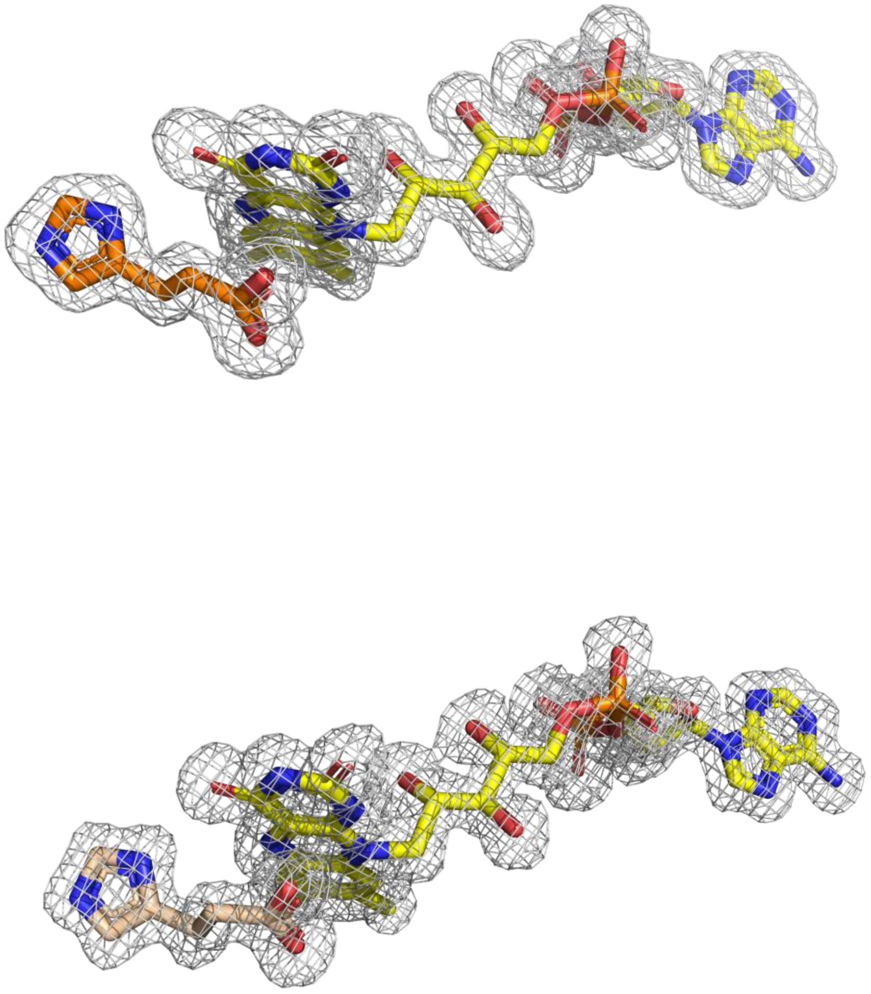

Supplementary Figure 2. Fo-Fc omit maps for the ligands (urocanate – upper panel in orange, imidazole propionate – lower panel in wheat, FAD – in yellow) and FAD at 3.0 sigma, carved at 1.4 Å.

The maps were generated using Polder maps in Phenix[1].

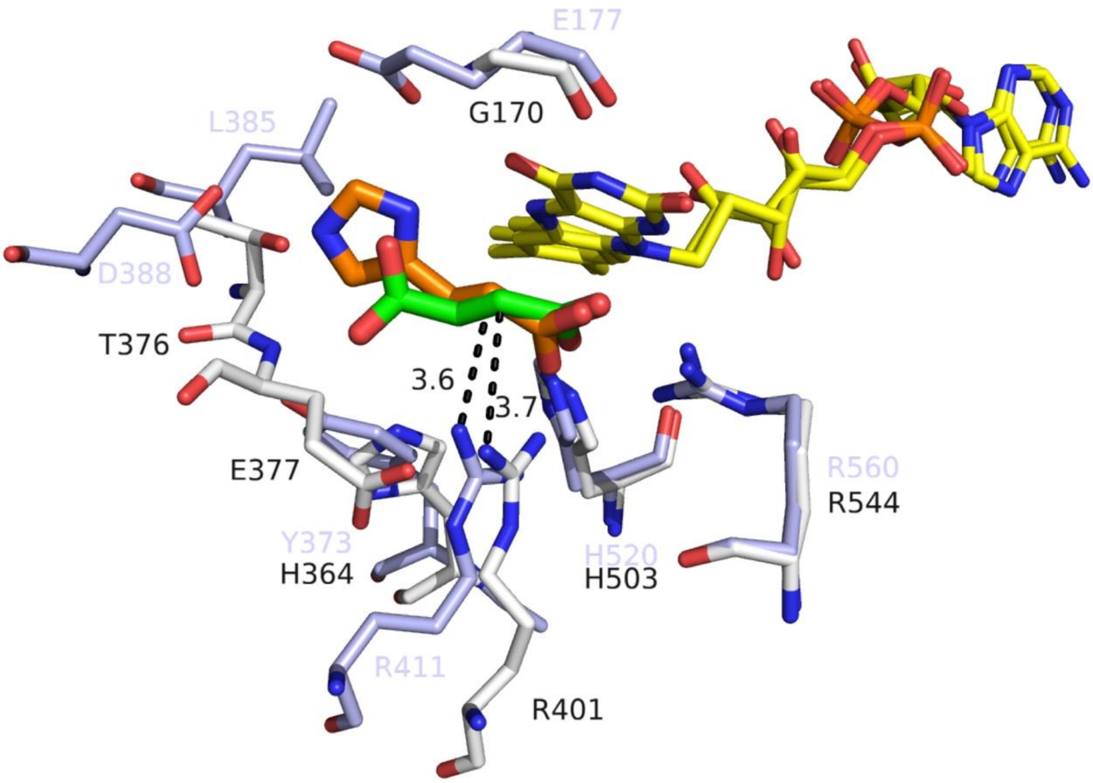

Supplementary Figure 3. Comparison of the active site between the uro-FAD (light blue) and the fumarate reductase (white) from *Shewanella putrefaciens* MR-1 (PDB ID: 1D4E). The distance from the Arg411/Arg401 to the substrate C2/C3 is indicated.

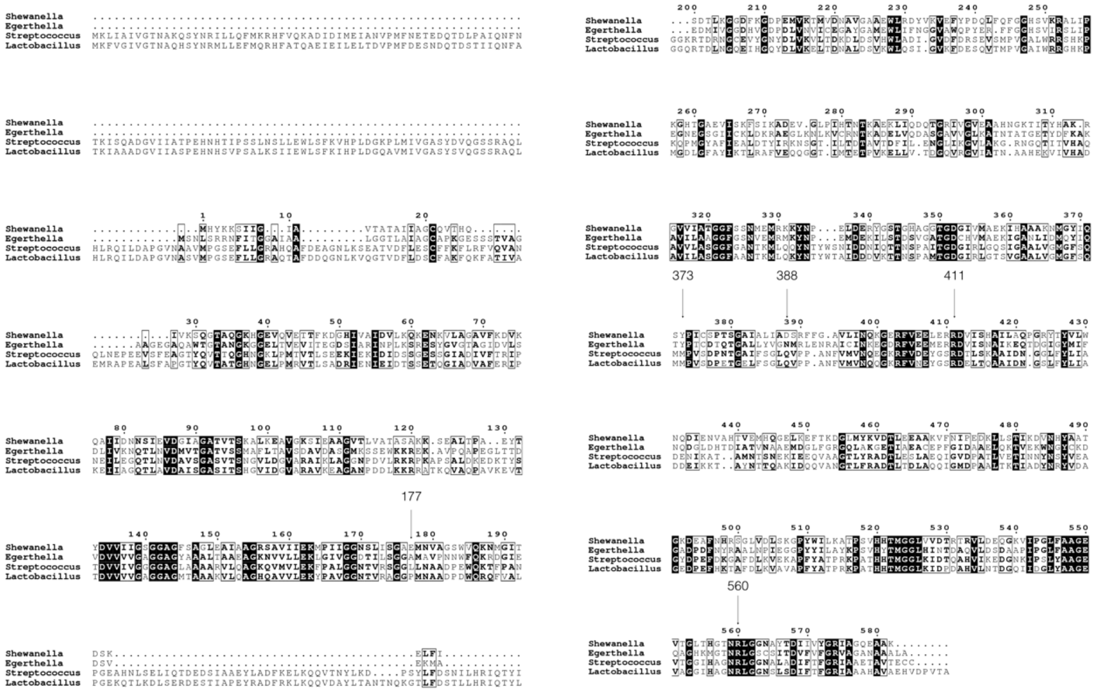

Supplementary Figure 4. Protein sequence alignment of UrdA from *Shewanella oneidensis* (uniprot ID Q8CVD0), *Egerthella lenta* (uniprot ID C8WLE3), *Streptococcus mutants* (uniprot ID Q8DW88) and *Lactobacillus plantarum* (uniprot ID A0A450RPI8). Binding site residues are indicated by arrows, *S. oneidensis* numbering. Alignment performed using EMBL-EBI server [2] Clustal Omega[3] and ESPript 3[4].

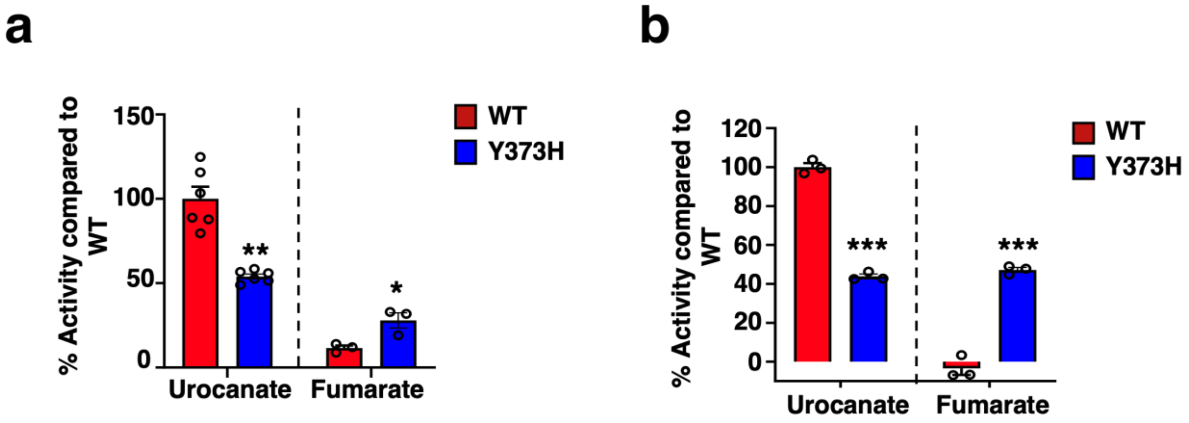

Supplementary Figure 5. The role of Y373 in substrate preference in (a) UrdA' and in (b) full-length UrdA. Relative urocanate or fumarate reductase activity was calculated compared to WT in the presence of 0.25 mM urocanate or 10 mM fumarate, respectively by using OD_{620} subtracted from controls (without substrate) at 5 min reaction. Data are mean ± s.e.m. **P* < 0.05, ***P* < 0.01, ****P* < 0.001. Urocanate reductase activity or fumarate reductase activity were independently compared between WT and Y373H with unpaired two-tailed Student's *t*-tests (a, b). n=6 independent experiments, for the activity towards urocanate (*P* value = 0.0008) and n=3 independent experiments for the fumarate reductase activity (*P* value = 0.0256) in (a). n=3 independent experiments for urocanate (*P* value <0.0001) and fumarate reductase activity (*P* value = 0.0002), respectively in (b). Source data are provided as a Source Data file.

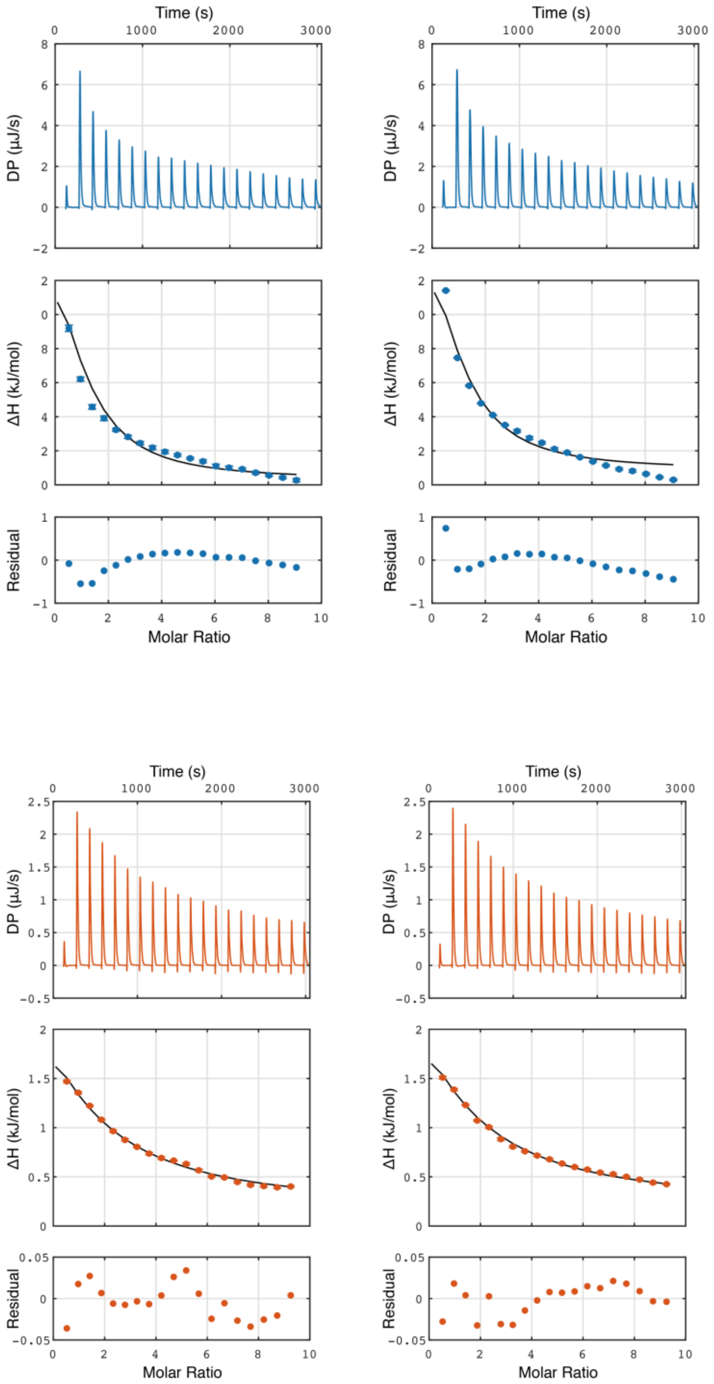

Supplementary Figure 6. ITC measurements for urocanate (blue) and imidazole propionate (orange) binding at UrdA’, showing the two data sets that form a triplicate together with the data shown in Fig. 2d of the main text. The full line shows the simultaneous fit to the three replicate data sets. Errors in individual ITC data points are estimated based on the baseline uncertainty, as implemented in NITPIC [5].

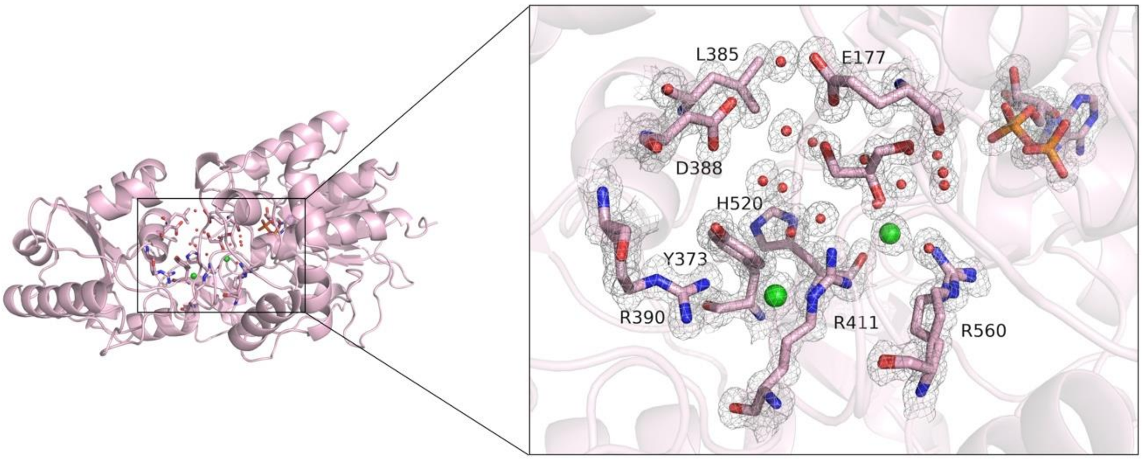

Supplementary Figure 7. Active site in the apo-ADP structure. Water molecules shown as red spheres and chloride ions as green spheres. 2mFo-DFc map contoured at 1σ.

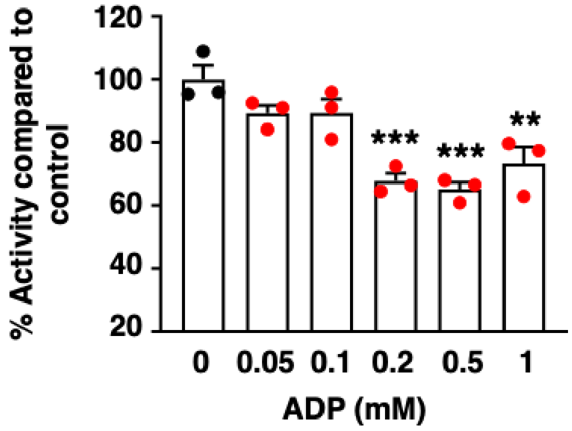

Supplementary Figure 8. ADP-induced inhibition of UrdA' activity. UrdA' activity assay was performed in the presence of 0.5 mM urocanate and varying concentrations of ADP (n =3, independent experiments). Data are mean ± s.e.m, ***P* < 0.01, ****P* < 0.001. One-way ANOVA with Dunnett's multiple comparisons test. Adjusted *P* values for 0 vs 0.05; 0 vs 1; 0 vs 0.2; 0 vs 0.5 and 0 vs 1 were 0.2089; 0.2145; 0.0002; 0.0001 and 0.0012 respectively. Source data are provided as a Source Data file.

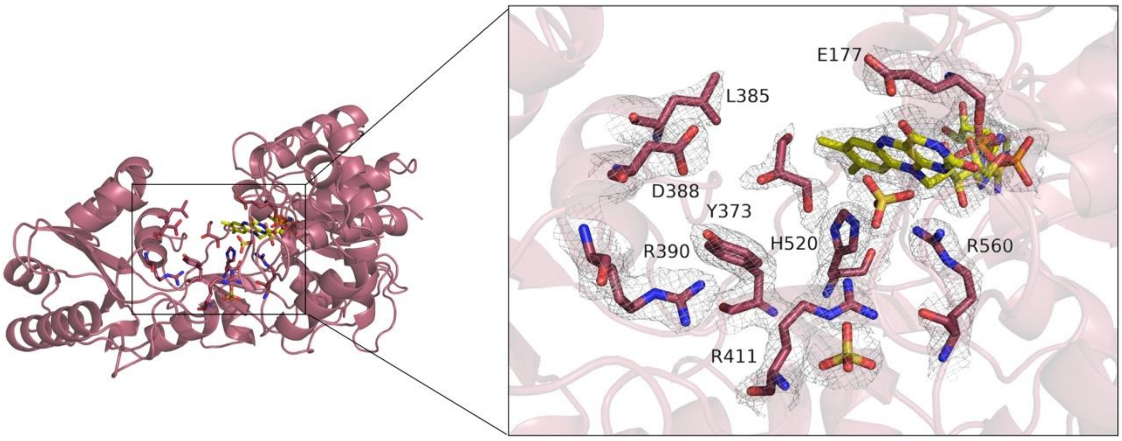

Supplementary Figure 9. Active site in the apo-FAD structure. 2mFo-DFc map contoured at 1σ.

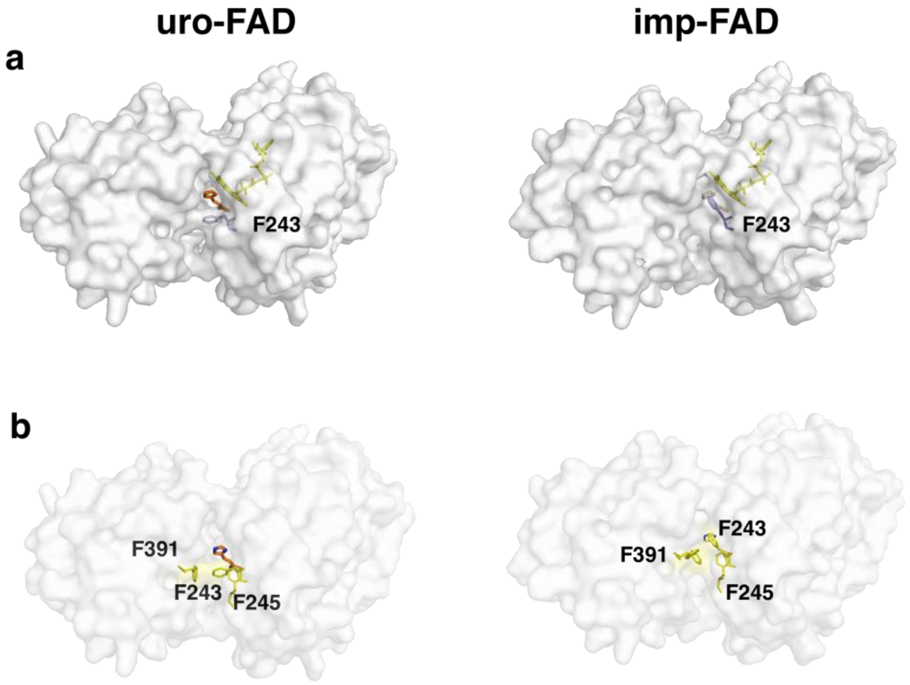

Supplementary Figure 10. The “hydrophobic lid” in urocanate and imidazole propionate bound structures. (a) F243 changes conformation when the product is bound – uro-FAD structure on the left and imp-FAD on the right in surface representation with F243 shown as sticks with urocanate (orange), ImP (wheat) and FAD (yellow). (b) Three phenylalanine residues comprise an important spot, which seemingly affects the substrate/product binding in the active site. Uro-FAD structure on the left and imp-FAD on the right in surface representation with F243, F245 and F391 shown as yellow sticks with urocanate (orange) and ImP (wheat).

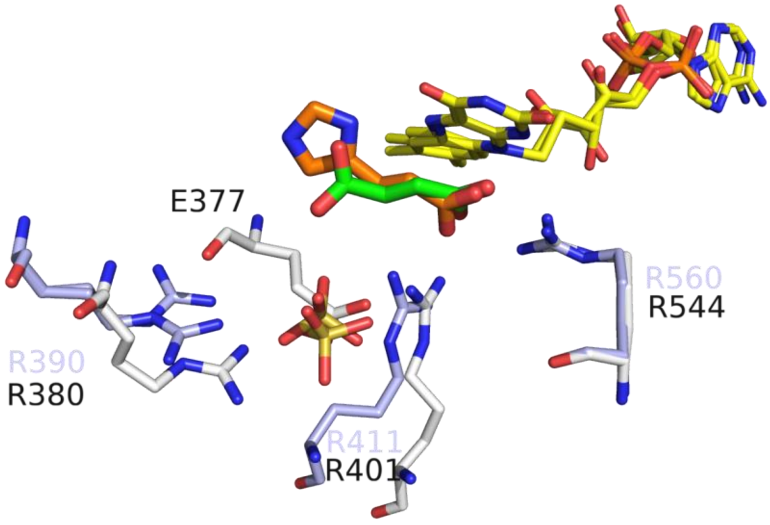

Supplementary Figure 11. A sulfate molecule observed in uro-FAD structure overlaps with an active site E377 residue in the fumarate reductase (white) from *Shewanella putrefaciens* MR-1 (pdb id 1D4E).

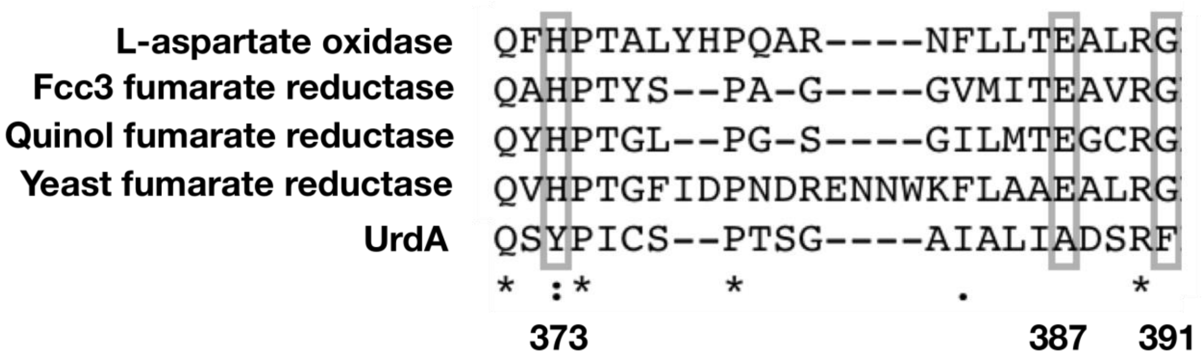

Supplementary Figure 12. UrdA can be distinguished from related enzymes by three residues – Y373, A387 and F391. To illustrate these differences the sequences of L-aspartate oxidase, Fcc3 fumarate reductase, quinol fumarate reductase, yeast fumarate reductase (protein sequence IDs retrieved from PDB database are 1CHU, 1D4E, 1L0V and 5ZYN respectively) and UrdA were aligned using T-Coffee method [6].

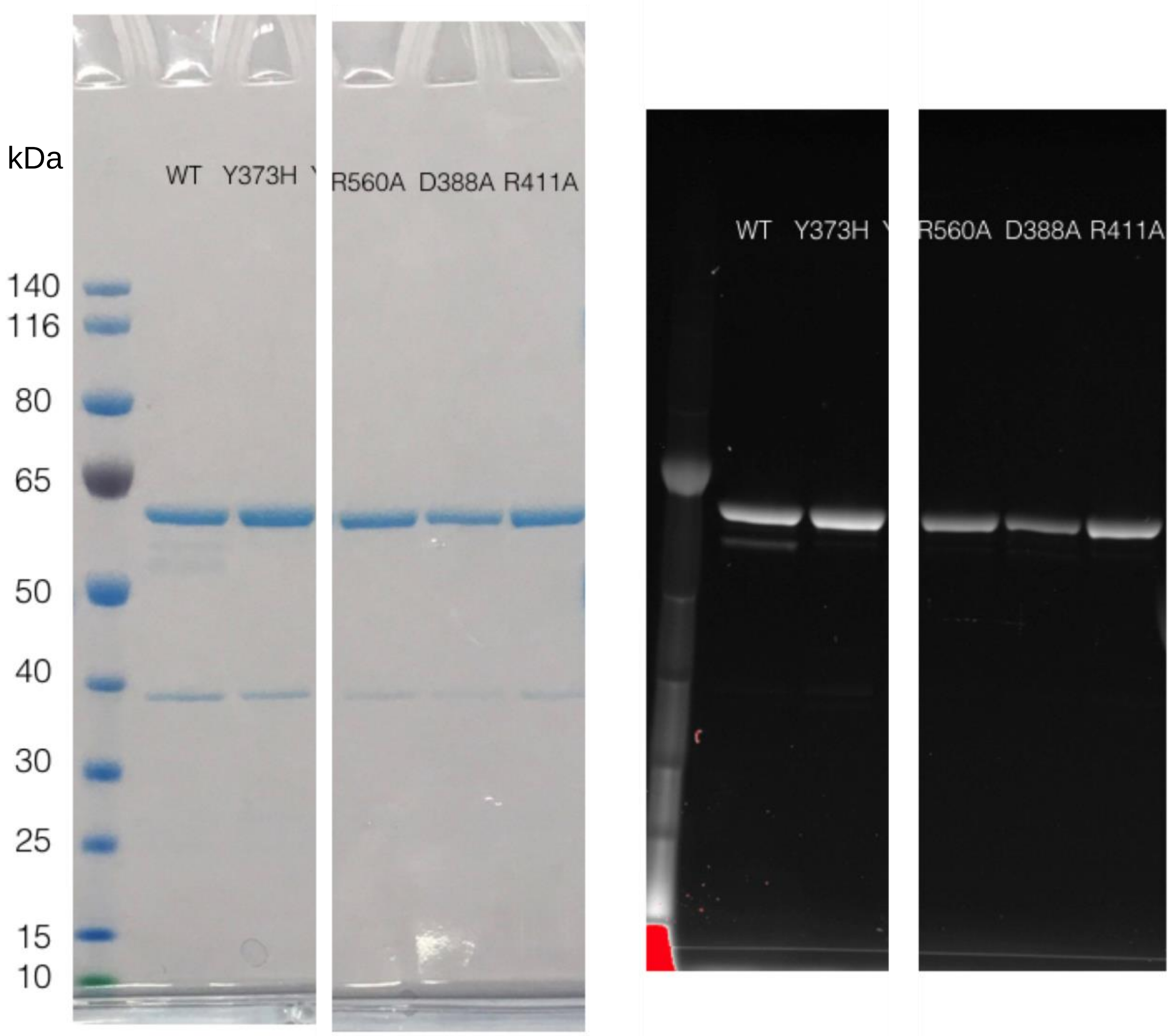

Supplementary Figure 13. SDS-PAGE of the purified UrdA variants after *in-vitro* flavinylation and subsequent size exclusion chromatography. Left – Coomassie stained gel, including the molecular weight ladder in kDa (PageRuler, ThermoFisher), right – the same gel exposed to a UV light prior staining.

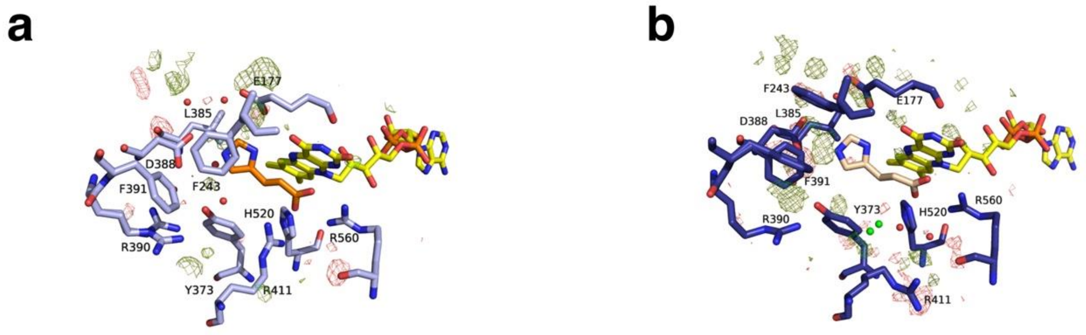

Supplementary Figure 14. Fo-Fc at sigma 3.0 for the (a) uro-FAD and (b) imp-FAD binding sites.

Supplementary References

1 Liebschner, D. *et al*. Polder maps: improving OMIT maps by excluding bulk solvent. *Acta Crystallogr D Struct Biol* **73**, 148-157, doi:10.1107/S2059798316018210 (2017).

2 Madeira, F. *et al*. The EMBL-EBI search and sequence analysis tools APIs in 2019. *Nucleic Acids Res* **47**, W636-W641, doi:10.1093/nar/gkz268 (2019).

3 Sievers, F. *et al*. Fast, scalable generation of high-quality protein multiple sequence alignments using Clustal Omega. *Mol Syst Biol* **7**, 539, doi:10.1038/msb.2011.75 (2011).

4 Robert, X. & Gouet, P. Deciphering key features in protein structures with the new ENDscript server. *Nucleic Acids Res* **42**, W320-324, doi:10.1093/nar/gku316 (2014).

5 Keller, S. *et al*. High-precision isothermal titration calorimetry with automated peak-shape analysis. *Anal Chem* **84**, 5066-5073, doi:10.1021/ac3007522 (2012).

6 Notredame, C., Higgins, D. G. & Heringa, J. T-Coffee: A novel method for fast and accurate multiple sequence alignment. *J Mol Biol* **302**, 205-217, doi:10.1006/jmbi.2000.4042 (2000).
