# Supplementary material for: Structural characterization of the microbial enzyme urocanate reductase mediating imidazole propionate production
Source: Nat Commun. 2021 Mar 1;12:1347. doi: 10.1038/s41467-021-21548-y (PMC7921117; doi:10.1038/s41467-021-21548-y)
Supplement: Supplementary file 3 — Reporting Summary [file 41467_2021_21548_MOESM3_ESM.pdf]

nature research

Corresponding author(s): Karin Lindkvist-Petersson

Last updated by author(s): Jan 19, 2021

Reporting Summary

Nature Research wishes to improve the reproducibility of the work that we publish. This form provides structure for consistency and transparency in reporting. For further information on Nature Research policies, see Authors & Referees and the Editorial Policy Checklist .

Statistics

For all statistical analyses, confirm that the following items are present in the figure legend, table legend, main text, or Methods section.

n/a	Confirmed	
☐	☒	The exact sample size (*n*) for each experimental group/condition, given as a discrete number and unit of measurement
☐	☒	A statement on whether measurements were taken from distinct samples or whether the same sample was measured repeatedly
☐	☒	The statistical test(s) used AND whether they are one- or two-sided<br>*Only common tests should be described solely by name; describe more complex techniques in the Methods section.*
☒	☐	A description of all covariates tested
☒	☐	A description of any assumptions or corrections, such as tests of normality and adjustment for multiple comparisons
☐	☒	A full description of the statistical parameters including central tendency (e.g. means) or other basic estimates (e.g. regression coefficient) AND variation (e.g. standard deviation) or associated estimates of uncertainty (e.g. confidence intervals)
☐	☒	For null hypothesis testing, the test statistic (e.g. *F*, *t*, *r*) with confidence intervals, effect sizes, degrees of freedom and *P* value noted<br>*Give P values as exact values whenever suitable.*
☒	☐	For Bayesian analysis, information on the choice of priors and Markov chain Monte Carlo settings
☒	☐	For hierarchical and complex designs, identification of the appropriate level for tests and full reporting of outcomes
☒	☐	Estimates of effect sizes (e.g. Cohen's *d*, Pearson's *r*), indicating how they were calculated

Our web collection on statistics for biologists contains articles on many of the points above.

Software and code

Policy information about availability of computer code

Data collection	X-ray data were collected at PETRA III P13 beamline at EMBL in Hamburg, Germany (apo-ADP structure) and at Paul Scherrer Institute, Villigen, Switzerland at the beamline X06SA (uro-FAD, imp-FAD and apo-FAD structures).
Data analysis	The X-ray data was processed in XDS (version Mar 15, 2019) and CCP4 (version 7.0.068, AIMLESS version 0.7.4 within CCP4) suite programs. PHENIX (version 14.2 and 1.15.2) was used for molecular replacement, model building and refinement in Phaser, AutoBuild and phenix.refine respectively within PHENIX. The structure models were build and edited in Coot (version 0.8.92). Structure figures prepared in Pymol 2.1. Chemical structures were drawn in Marvin Sketch (version 19.12 ChemAxon). Protein domain movements calculdarted using an online server DynDom (http://dyndom.cmp.uea.ac.uk). ITC data analysis was performed using MATLAB (version R2017b) and NITPIC (version 1.2.2).

For manuscripts utilizing custom algorithms or software that are central to the research but not yet described in published literature, software must be made available to editors/reviewers. We strongly encourage code deposition in a community repository (e.g. GitHub). See the Nature Research guidelines for submitting code & software for further information.

Data

Policy information about availability of data

All manuscripts must include a data availability statement. This statement should provide the following information, where applicable:

- Accession codes, unique identifiers, or web links for publicly available datasets
- A list of figures that have associated raw data
- A description of any restrictions on data availability

The four X-ray structures (coordinates and structure factors) have been deposited to the PDB with the following accession numbers: 6T85 for apo-ADP, 6T86 for apo-FAD, 6T87 for uro-FAD and 6T88 for imp-FAD. Materials are available upon reasonable request. Source data are provided with this paper underlying Figs 1a, 2a–d and Supplementary Figs 5a-b, 6 and 8.

Field-specific reporting

Please select the one below that is the best fit for your research. If you are not sure, read the appropriate sections before making your selection.

- [x] Life sciences
- [ ] Behavioural & social sciences
- [ ] Ecological, evolutionary & environmental sciences

For a reference copy of the document with all sections, see nature.com/documents/nr-reporting-summary-flat.pdf

Life sciences study design

All studies must disclose on these points even when the disclosure is negative.

Sample size	Sample size determination was not used. Enzyme activity assays and ITC assays were performed on purified recombinant protein.
Data exclusions	For the apo-FAD dataset, 2000 frames were collected, however 800 frames were excluded from processing as this would result in bad merging statistics, likely due to radiation damage or poor area of the crystal.<br>One data point is missing for the UrdA'R560A activity in Fig. 2a due to its negative value but is provided in a Source Data file.
Replication	In vitro enzymatic activity measurements were performed in replicates of 3 or 5 for each different protein variant (2-domain and full-length WT and the mutants). Similar results were achieved for the WT and corresponding mutant variants of the truncated and the full-length proteins. ITC measurements were performed three times, using purified 2-domain WT enzyme. The results were deemed reliable due to little variation between the replicates.
Randomization	Randomization is not applicable as no group allocation was used.
Blinding	Blinding is not applicable as no group allocation was used.

Reporting for specific materials, systems and methods

We require information from authors about some types of materials, experimental systems and methods used in many studies. Here, indicate whether each material, system or method listed is relevant to your study. If you are not sure if a list item applies to your research, read the appropriate section before selecting a response.

Materials & experimental systems

n/a	Involved in the study	
x		Antibodies
x		Eukaryotic cell lines
x		Palaeontology
x		Animals and other organisms
x		Human research participants
x		Clinical data

Methods

n/a	Involved in the study	
x		ChIP-seq
x		Flow cytometry
x		MRI-based neuroimaging
